# Supplementary material for: Combined Toxic Effects of Lead and Glyphosate on Apis cerana cerana
Source: Insects. 2024 Aug 27;15(9):644. doi: 10.3390/insects15090644 (PMC11432057; doi:10.3390/insects15090644)
Supplement: Supplementary file 1 [file insects-15-00644-s001.zip › insects-3167197-supplementary.pdf]

Table S1. The Chi2 values of the goodness-of-fit test for Probit regression are presented together with the slope of the regression ± SE.

**Exposure time (h)**	**Glyphosate alone**			**Lead chloride alone**			**Glyphosate combined**			**Lead chloride combined**			**Additive index (AI)**
	chi2	SLOPE	SE	chi2	SLOPE	SE	chi2	SLOPE	SE	chi2	SLOPE	SE	
72	6.6	0.0002	0.10	6.7	0.01	0.12	8.8	0.01	0.11	6.2	0.02	0.14	0.43
96	8.1	0.0003	0.96	7.1	0.02	0.14	5.5	0.01	0.12	8.8	0.03	0.15	0.30

Note: All df=6.
